# Supplementary material for: HIVprotI: an integrated web based platform for prediction and design of HIV proteins inhibitors
Source: J Cheminform. 2018 Mar 9;10:12. doi: 10.1186/s13321-018-0266-y (PMC5845081; doi:10.1186/s13321-018-0266-y)
Supplement: Supplementary file 2 — Additional file 2. Source code of HIVProtI web server. [file 13321_2018_266_MOESM2_ESM.zip › HIVprotI_Source-code/hivprotI/weka-3-6-6/doc/weka/core/tokenizers/WordTokenizer.html]

WordTokenizer


---


|  |  |  |
| --- | --- | --- |
| **Overview** \| **Package** \| **Class** \| **Tree** \| **Deprecated** \| **Index** \| **Help** \| **Weka's home** | |  |
| **PREV CLASS**   NEXT CLASS | **FRAMES**    **NO FRAMES**     **All Classes** |  |
| SUMMARY: NESTED \| FIELD \| CONSTR \| METHOD | DETAIL: FIELD \| CONSTR \| METHOD |  |


---


## weka.core.tokenizers Class WordTokenizer

```
java.lang.Object
  weka.core.tokenizers.Tokenizer
      weka.core.tokenizers.CharacterDelimitedTokenizer
          weka.core.tokenizers.WordTokenizer
```

**All Implemented Interfaces:**: java.io.Serializable, java.util.Enumeration, OptionHandler, RevisionHandler

---

``` public class WordTokenizer extends CharacterDelimitedTokenizer ```

A simple tokenizer that is using the java.util.StringTokenizer class to tokenize the strings.


Valid options are:

```
 -delimiters <value>
  The delimiters to use
  (default ' \r\n\t.,;:'"()?!').
```

**Version:**
:   $Revision: 1.4 $

**Author:**
:   FracPete (fracpete at waikato dot ac dot nz)

**See Also:**: Serialized Form

---

**Constructor Summary**

`WordTokenizer()`


| **Method Summary** | |
| --- | --- |
| `java.lang.String` | `getRevision()`             Returns the revision string. |
| `java.lang.String` | `globalInfo()`             Returns a string describing the stemmer |
| `boolean` | `hasMoreElements()`             Tests if this enumeration contains more elements. |
| `static void` | `main(java.lang.String[] args)`             Runs the tokenizer with the given options and strings to tokenize. |
| `java.lang.Object` | `nextElement()`             Returns the next element of this enumeration if this enumeration object has at least one more element to provide. |
| `void` | `tokenize(java.lang.String s)`             Sets the string to tokenize. |

**Methods inherited from class weka.core.tokenizers.CharacterDelimitedTokenizer**

`delimitersTipText, getDelimiters, getOptions, listOptions, setDelimiters, setOptions`

**Methods inherited from class weka.core.tokenizers.Tokenizer**

`runTokenizer, tokenize`

**Methods inherited from class java.lang.Object**

`equals, getClass, hashCode, notify, notifyAll, toString, wait, wait, wait`

**Constructor Detail**

### WordTokenizer

```
public WordTokenizer()
```


**Method Detail**

### globalInfo

```
public java.lang.String globalInfo()
```

:   Returns a string describing the stemmer

    :   **Specified by:**: `globalInfo` in class `Tokenizer`
    :   **Returns:**: a description suitable for displaying in the explorer/experimenter gui

---


### hasMoreElements

```
public boolean hasMoreElements()
```

:   Tests if this enumeration contains more elements.

    :   **Specified by:**: `hasMoreElements` in interface `java.util.Enumeration` **Specified by:**: `hasMoreElements` in class `Tokenizer`
    :   **Returns:**: true if and only if this enumeration object contains at least one more element to provide; false otherwise.

---


### nextElement

```
public java.lang.Object nextElement()
```

:   Returns the next element of this enumeration if this enumeration object
    has at least one more element to provide.

    :   **Specified by:**: `nextElement` in interface `java.util.Enumeration` **Specified by:**: `nextElement` in class `Tokenizer`
    :   **Returns:**: the next element of this enumeration.

---


### tokenize

```
public void tokenize(java.lang.String s)
```

:   Sets the string to tokenize. Tokenization happens immediately.

    :   **Specified by:**: `tokenize` in class `Tokenizer`
    :   **Parameters:**: `s` - the string to tokenize

---


### getRevision

```
public java.lang.String getRevision()
```

:   Returns the revision string.

    :   **Returns:**: the revision

---


### main

```
public static void main(java.lang.String[] args)
```

:   Runs the tokenizer with the given options and strings to tokenize.
    The tokens are printed to stdout.

    :   **Parameters:**: `args` - the commandline options and strings to tokenize


---


|  |  |  |
| --- | --- | --- |
| **Overview** \| **Package** \| **Class** \| **Tree** \| **Deprecated** \| **Index** \| **Help** \| **Weka's home** | |  |
| **PREV CLASS**   NEXT CLASS | **FRAMES**    **NO FRAMES**     **All Classes** |  |
| SUMMARY: NESTED \| FIELD \| CONSTR \| METHOD | DETAIL: FIELD \| CONSTR \| METHOD |  |


---
